# Understanding health management and safety decisions using signal processing and machine learning

**DOI:** 10.1186/s12874-019-0756-2

**Published:** 2019-06-13

**Authors:** Lisa Aufegger, Colin Bicknell, Emma Soane, Hutan Ashrafian, Ara Darzi

**Affiliations:** 1 Patient Safety Translational Research Centre, Imperial College London, London, UK; 2 Department of Management, London School of Economics and Political Science, London, UK

## Abstract

**Background:**

Small group research in healthcare is important because it deals with interaction and decision-making processes that can help to identify and improve safer patient treatment and care. However, the number of studies is limited due to time- and resource-intensive data processing. The aim of this study was to examine the feasibility of using signal processing and machine learning techniques to understand teamwork and behaviour related to healthcare management and patient safety, and to contribute to literature and research of teamwork in healthcare.

**Methods:**

Clinical and non-clinical healthcare professionals organised into 28 teams took part in a video- and audio-recorded role-play exercise that represented a fictional healthcare system, and included the opportunity to discuss and improve healthcare management and patient safety. Group interactions were analysed using the recurrence quantification analysis (RQA; Knight et al., 2016), a signal processing method that examines stability, determinism, and complexity of group interactions. Data were benchmarked against self-reported quality of team participation and social support. Transcripts of group conversations were explored using the topic modelling approach (Blei et al., 2003), a machine learning method that helps to identify emerging themes within large corpora of qualitative data.

**Results:**

Groups exhibited stable group interactions that were positively correlated with perceived social support, and negatively correlated with predictive behaviour. Data processing of the qualitative data revealed conversations focused on: (1) the management of patient incidents; (2) the responsibilities among team members; (3) the importance of a good internal team environment; and (4) the hospital culture.

**Conclusions:**

This study has shed new light on small group research using signal processing and machine learning methods. Future studies are encouraged to use these methods in the healthcare context, and to conduct further research on how the nature of group interaction and communication processes contribute to the quality of team and task decision-making.

**Electronic supplementary material:**

The online version of this article (10.1186/s12874-019-0756-2) contains supplementary material, which is available to authorized users.

## Background

Today’s healthcare service is increasingly dependent on interdisciplinary teamwork in order to provide holistic and patient-centred care [1]. Effective teamwork entails independent yet collaborative work commitment toward a shared goal, where each member values and relies on each other’s expertise, roles and responsibilities [2]. Such collaboration increases the quality of decision-making and the effectiveness of performance outcomes because each member contributes through their unique expertise and specialism [3]. To achieve team cohesion and consensus in decision-making, members engage in discrete stages of knowledge processing, coordination, and integration, which are manifested in non-linear and dynamic group interactions, and observed in behaviour such as information exchange, evaluation and systematization [4–7].

Small group research, which is embedded in the wider context of social psychology [8], focuses on such group dynamics. In this setting, the term “groups” refers to social, collective entities that have a shared and common purpose. This sense of shared purpose is expressed through coordinated task activities and within environments in which participants work together to accomplish a goal while experiencing a feeling of togetherness. Based on this concept, studies of small groups typically explore aspects of group related to formation and development, group structures, group communication and group decision-making [9, 10].

Group interactions are usually addressed through the systematic coding of occurrence of behaviour on a predetermined scale either in situ, from video, or both [8]. Alternatively, interactions can also be examined through retrospective qualitative analysis [8]. The results, such as the frequency and duration of specific behaviours and emerging themes from the team members’ discourses, are generally presented and discussed descriptively [11, 12], or analysed using more advanced statistical methods such as general linear models, a predictive technique suitable for both multiple qualitative and quantitative variables [13, 14].

Observational and qualitative data provide valuable insights into the contextual and temporal features of human interactions that can be found in team meetings. However, when it comes to large corpora of data, each method has limitations [15]. For instance, while observational data of team behaviour provides useful information about social interactional processes*,* the analytical process is costly in terms of time and resources [16]. Qualitative research, while rich in information, may be influenced by personal biases (i.e. researcher’s opinion and viewpoint in the topic analyzed) [17], is extremely time-consuming and, as such, is usually only feasible for small-scale studies.

One way to extract meaningful and unbiased information, and to overcome the challenges and limitations associated with resource-intensive analyses, is the application of signal processing and statistical machine learning (ML) techniques [18, 19]. The former allows for processing continuous, non-linear information from physical observation [20], including the examination of physiological signals such as breathing or heart rate variability. The latter approach, ML techniques, identifies optimal parameters to capture patterns and regularities in a given system, such as are needed in risk assessment and document classification [21]. In small group research, both have been proposed as novel techniques to address relationship management in group conversations in order to understand group characteristics, make predictions, or both [22] (for a detailed review, see Gatica-Perez [23]).

### Context

**Fig. 1:**
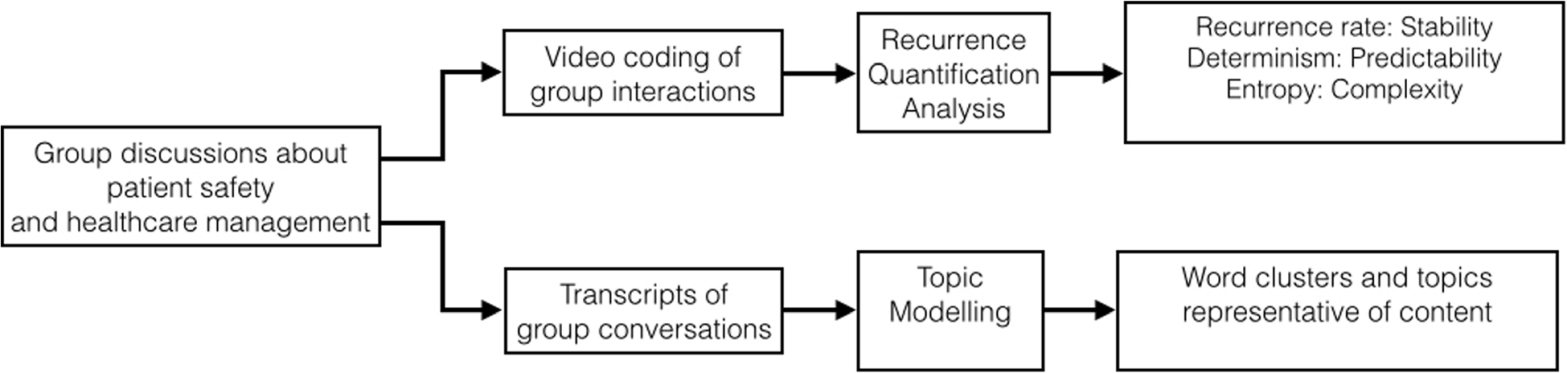
Flowchart of processing techniques applied for both quantitative and qualitative data sets

This study examined group interactions and communication processes related to healthcare management and patient safety using a role-play exercise. The results were considered in relation to self-reported quality of teamwork participation and social support [3]. This was done by utilising two methods drawn from engineering. First, we applied recurrence quantification analysis, a signal processing method that examines stability, determinism, and complexity of group interactions; and the topic modelling approach, a machine learning method that helps to identify emerging themes within large corpora of qualitative data (cf. Figure 1). Stability refers to the recurrence of interaction between members, while determinism reflects a predictive constellation of group interactions over time. Lastly, complexity is the degree to which the interactions are “chaotic” and follow a disorderly pattern. Thus, we examined the relationship between self-reported quality of participation and social support and the RQA in response to the role-play exercise.

Second, we used the topic modelling approach to understand whether it is a feasible approach to analysis small group conversations. We also examined whether the role-play exercise provides an environment that allows for a naturalistic group processes related to content addressing healthcare management and patient safety. As illustrated in Fig. 1, the RQA was applied to data extracted and coded from video recordings, while the topic modelling approach was used to explore the audio-recorded transcripts of group discussions.

### Recurrence quantification analysis

**Fig. 2:**
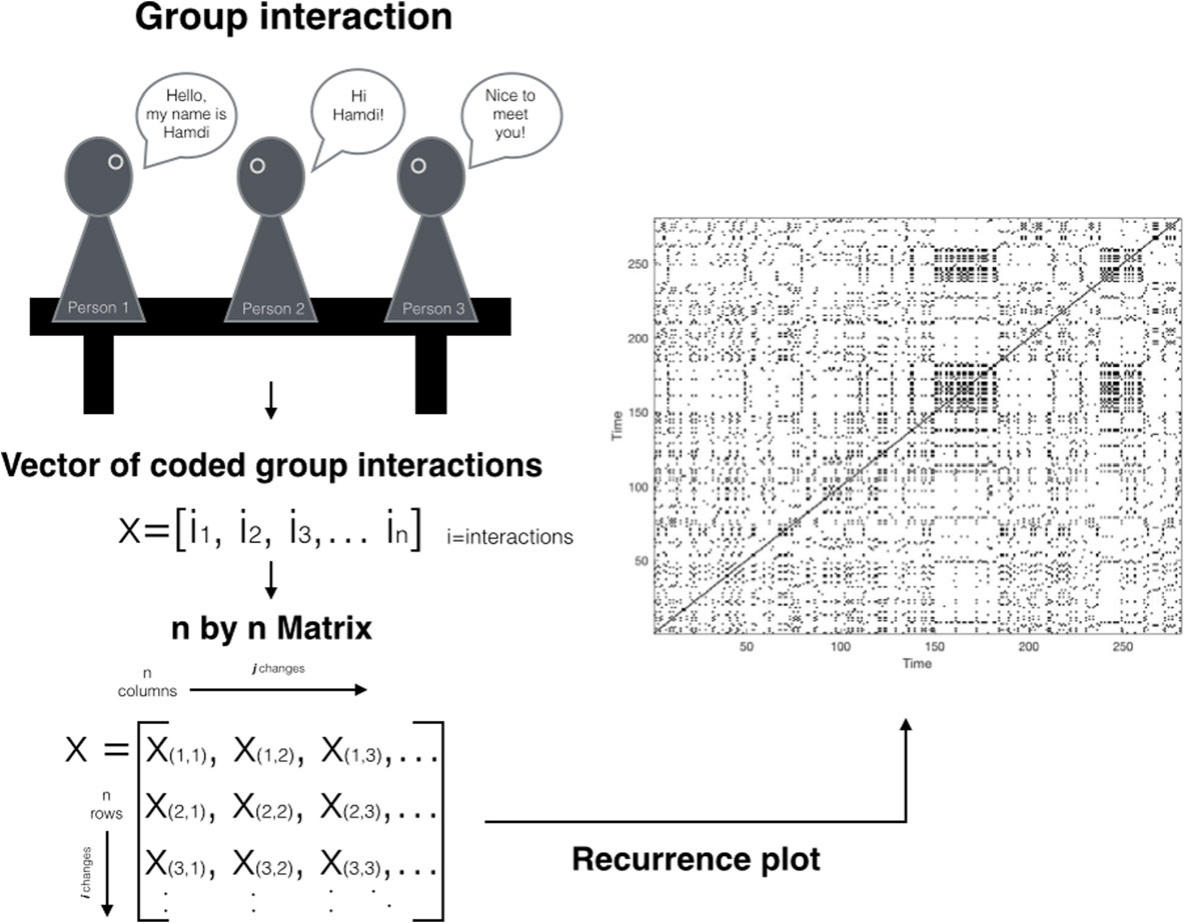
Example of a recurrence plot. The black dots represent recurrent states within a given time series

Originally designed as a graphical technique, the RQA assesses system dynamics found in discrete time series (e.g. physical signals such as heart rate variability) by providing a visual representation and an index of the recurrence, predictability, and complexity of states at different points in time [24]. This is achieved by creating a matrix of a given length of a time series x, where the recurrence plot, if the value of x at time point (i) is sufficiently close (within a given threshold) to the value of x at time point (j), displays a dot plotted at x(i,j) [25]. In the example illustrated in Fig. 2, time series data for groups are ordered to a vector of sequences of group interactions over time, before being transformed into a recurrence plot. Each recurrent point is represented by a black dot in the recurrence plot, with a main diagonal that consists of coded data who’s row number and column number are equal (x_j,j_).

The matrix of pairwise combinations x(i,j) can then be assessed in terms of its structural complexity of diagonal lines by extracting three complexity measures from the RQA: (1) the recurrence rate, (2) the percentage of determinism; and (3) the entropy [26, 27].

The recurrence rate is a density measure that indicates the stability of a system by assessing how often the system revisits a past state in relation to the total number of possible recurrences. As illustrated in the recurrence plot (Fig. 2), the proportion of black dots represents the points of recurrent states. For instance, a recurrence rate of 30 indicates that a specific state was revisited a third of the time series. Recurrence rate can range from 0 to 100, the former indication no recurrence at all and the latter a continuous repetition of the same state [28, 29].

The percentage of determinism informs about the predictability of the system based on a pre-defied minimum length of recurrent points [30]. As can be observed in Fig. 2, upward-running lines represent periods when a system exhibits identical states over time. A plot that has only few diagonal lines suggests a random dynamic process. Levels of predictability can range from 0%, classified as random system, to 100%, referring to as highly deterministic system.

Entropy is a complexity measure that describes the complexity of the distribution probability of diagonal lines of the same length [31, 32]. In other words, the entropy provides information on the degree of perplexity or uncertainty of sequences of group interactions over time. Groups that exhibit disordered or chaotic interactions between team members should exhibit a higher degree of entropy than groups that do not. Theoretically, the entropy should be zero for matrixes with diagonals of the exact lengths, positive for matrixes with diagonals of many different lengths, and infinite for matrixes with diagonals of random lengths [30].

In social science, applications of these tools are still in their infancy. For instance, the majority of RQA studies in small group research has investigated reoccurring coupling effects in physiological (e.g. cardiac inter-beat intervals) and behavioural (e.g. postural sway) signals during mentally challenging tasks [33], or investigated the degree of synchrony in behaviour (e.g. head movements) between participants during positive versus negative conversations, and with and without informative visual stimuli [34]. Similar to our study, two studies have investigated changes related to stability, determinism, and complexity of sequences of group interactions [28, 35]. Findings suggest that group members collaborating over multiple sessions develop a greater level of determinism of group interactions than groups that change membership each time, and that an increased level of predictability is associated with less flexibility in coordination and communication processes. However, none of these studies examined results in relation to objective or self-reported measures of teamwork behaviour and quality, respectively. Thus, further work is needed to understand the value of the RQA as a quality index in the context of teamwork in general, and there are specific applications to team processes that relate to healthcare management and patient safety.

### Topic modelling

Topic modelling is an optimisation process that allows for identifying co-occurring words and clusters of reoccurring patterns that appear together in the transcript [36, 37]. Similar to a factor analysis, words of high probability within an identified cluster represent semantically related content (e.g. leader, member, team, group). A commonly used and well-established topic modelling approach is the Latent Dirichlet Allocation (LDA), an unsupervised method (i.e. using unlabelled data), appropriate for the analysis of qualitative data analysis carried out in an exploratory fashion. For the LDA results to be meaningful, pre-processing steps have to be undertaken [38] such as, erasing punctuations, converting the text data to lowercase, tokenisation (i.e. sequences of characters are grouped together to allow for useful semantic units for processing) [39], removing a list of stop words such as “of”, “a”, “and”, removing words with two or fewer characters, and words with 15 or greater characters, and normalising words using the Porter [40] stemmer. The Porter stemmer removes various suffixes –ed, −ing, −ion, or –ions and leave the single term to be processed. For instance, talks, talking, talked will be reduced to the word stem “talk”. This is done to reduce the overall size of the vocabulary and computational burden, as well as to enhance the interpretability of results [38].

Next, LDA tests for a range of suitable numbers of topics per group, and cross-validates each model (i.e. number of topic) on a sub-set of documents (i.e. this is called training data, which was 10% of randomly selected partition within the data set, also called test data) [41]. The number of topics with the lowest degree of perplexity (i.e. the least complex model for the best prediction of the data) is then selected on the remaining data. The perplexity measure is similar to a confirmatory factor analysis, which uses a scree plot and the degree of Eigenvalue to assess and make a cut-off between relevant and non-relevant factors [42]. For defining the number of topics that best represent the data, the lowest degree of perplexity is determined by comparing the fit of LDA models of various numbers of topics on held-out documents (i.e. test data), and by visually inspecting the perplexity plot for each model. For instance, a perplexity plot could be generated for LDA models of 20, 40, 80, etc. numbers per topics. Here, the choice of number topics will depend the size and set up of data (e.g. abstracts versus twitter data versus full conversations). A greater model power is represented in a lower perplexity, indicating that there is less uncertainty about the documents [43]. Final results allow for the identification of (1) the number of topics for the whole set of data; and (2) the words within documents to specific topics.

We carried out the LDA using MATLAB, a mathematical computing software for engineers and scientists. Developed and published by Steyver’s and Griffith [44], the authors provide a Topic Modeling Toolbox that allows conducting an LDA – including the aforementioned pre-processing steps – in order to extract meaningful information. For this, no advanced or programming skills or complex and time-intensive manual pre-processing work is required.^1^

Previous studies have demonstrated that topic modelling is suitable for examining information from blogs, journal entries or open-ended responses in electronic health records for the purpose of e.g. opinion analysis [38, 45, 46]. However, no documentation exists whether the ML technique is sufficient in exploring and understanding small group conversations related to patient safety and healthcare management. Group conversations are rich in terms of complexity of content and language used. Thus, exploring whether findings from this study align with previous qualitative studies related to healthcare management and patient safety would support the application of the ML technique to this context.

## Methods

### Sample

Participant recruitment took place through the offices of a UK hospital and a UK university that offers executive education to healthcare professionals between November 2016 and December 2017. Potential participants were informed about the schedule of events and the time commitment required. The sample comprised 103 participants (28 groups of 3–5 participants; *mean age* = 33.01, *SD* = 8.53; *female* = 55) clinical and non-clinical managerial students (e.g. medicine students as well as executive students who have already obtained a professional qualification; *n* = 28) and healthcare professionals (nurses, surgical registrars, public health managers; *n* = 75) from a number of organisations that participants represented.

### Setting

The role-play exercise was built on a fictional healthcare system that contained recognisable issues, problems and opportunities to improve patient safety. Participants were asked to read through a portfolio of information that consisted of a description of the fictional healthcare trusts (e.g. in-patient and out-patient activity), reports on recent patient safety events, including a wrong lens implant, a forgotten swab after surgery and a medication error, and a booklet on effective practices related to teamwork, shared leadership and joint decision-making. The room for the role-play exercise included a table, up to 6 chairs and two video cameras positioned in the corner opposite to each other. The exercise lasted approximately 2 h. Activities were audio (Olympus WS-853) and video (2 GoPro4Black; setting 1080p wide at 60 frames per second) recorded, and observed by a member of the research team. Data collection took place at the offices of the research team.

The exercise was divided into three 30-min activities including an *individual* task, in which participants familiarised themselves with the material, a *group* task, where participants discussed how to improve patient safety and healthcare management, and a *debrief* in which a member of the research team provided participants with feedback on their teamwork skills. This study focuses on the data gathered during the group task.

### Group task

During the 30-min group task, participants were assigned a role that represented a Healthcare Management Committee, consisting of the Lead Medical Director, the Deputy Medical Director, the Chief Nurse, the Human Resource Manager, and the Finance Director. These roles were chosen because they are typically represented board level meeting related to healthcare management and patient safety. The overall task was to discuss and develop a list of recommendations to improve healthcare management and patient safety.

### Questionnaires

Before the group activity, participants completed a background questionnaire including sex, age and number of years of supervisory experience. After the group activity, participants rated their perceived team member’s ability to participate and provide input, and whether team members supported everyone actively participating. This was assessed by two sub-scales – quality of participation (i.e. voice) and social support – that were adapted from Carson et al.’s [2] internal team environment scale, a tool that examines the degree of team members’ perceived ability to encourage participation and support. Sample items are: “My team supports everyone actively participating in decision-making” and “Everyone on this team has a chance to participate and provide input.” Rated on a 7-point Likert scale (1 = “Strongly disagree” to 7 = “Strongly agree”), higher scores indicate a greater degree of perceived quality of participation. The Cronbach’s Alpha for the quality of participation was .85 and the value for social support was .81 [47].

## Data collection and processing

**Fig. 3:**
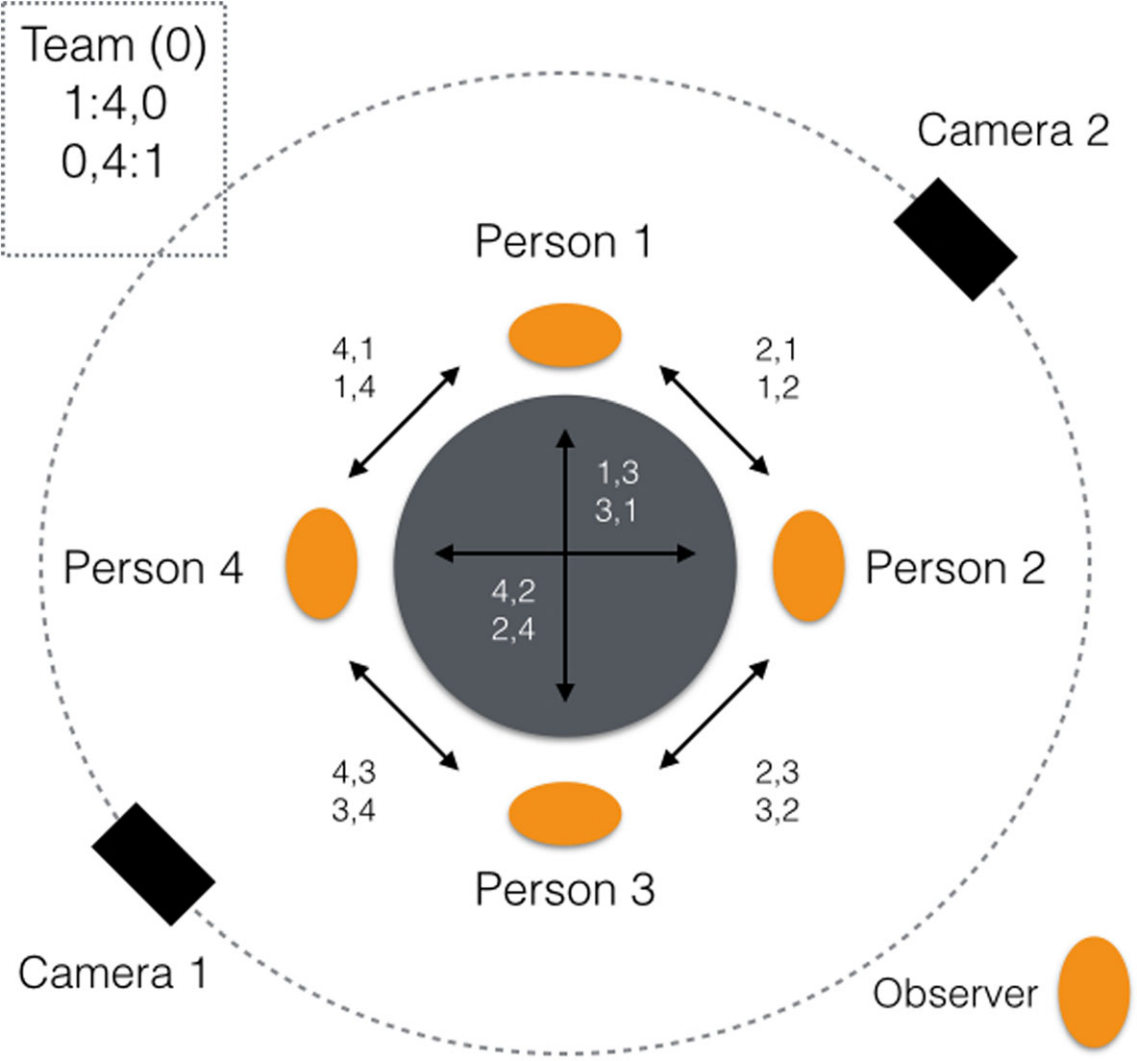
Layout of the room where the role-play exercise took place, as well as coding strategy for each team members and the team, respectively

In order to be able to appropriately investigate the characteristics of group processes and communication in the context of healthcare management and patient safety decisions, and in alignment with previous measures in small group research, three types of data were collected [8, 48]: First, we extracted and coded sequences of group interactions between members from the video recording (cf. Figure 3 of the layout of the room and the coded data). For this, time series data for each group were ordered to a vector of sequences of group interactions over time, before being transformed into a recurrence plot. The sequences were defined by the interaction between team members. Specifically, each member was assigned a number (1,2,3, etc.), with interactions being reflected in the number of dyadic combinations (e.g. 1–2, 2–3, 3–1, etc.). We also acknowledged the interaction between a team member and other team members; e.g. a member asking the other team members whether they approve of a suggestion and the remaining team members responding simultaneously, e.g. by agreeing. In these cases, an additional number was assigned to account for member-team interactions.

The interactions were categorised according to Bales’ Interaction Process Analysis (IPA) model. The IPA is a robust method for categorising systems of interaction in small face-to-face groups [49]. Behaviours were examined based on a predetermined a set of 12 behaviours that represent two categories: “social-emotional” and “task” (for more details, see Bales [50]). We coded videos using the MANGOLD INTERACT software [51], a video analysis tool that allows synchronised viewing, content coding, and event logging analysis of video recordings for observational research. Each video was analysed by two independent researchers. If behaviour was not visibly seen in one of the video recordings, the second video was used for clarification purposes. The consistency of the inter-rater agreement of frequency in behaviour and, thus, interactions was assessed by intra-class correlation coefficient 2-way random-effects model. The results showed a high degree of reliability between the two raters (*ICC* = .94). Following the coding of these interactions, we assessed the extent to which the stability, determinism, and complexity of sequences of group interactions. To establish the degree of determinism, and in alignment with Knight et al. (2016) [28] who also evaluated group interactions in simulated settings, the minimal length to account for deterministic interactions were set to two recurrent data points of sequences of group interactions over time.

Second, we audio-recorded group discussions that were transcribed by an external company for an agreed fee. For the transcription, a verbal intelligent style was applied. ‘Intelligent verbatim’ transcripts omit ‘ums’ and ‘ers’ and other fillers [52] to produce an accurate transcript which is also readable. Each team member’s input was transcribed line by line. This led to a total final word count of 53,485 words for all transcripts. Next, the transcripts were imported into MATLAB and pre-processing of the data was executed.

Third, self-reports on the team’s perceived quality of participation and social support. Self-reports were based on Carson et al.’s [2] internal team environment scales.

In summary, both observational data and transcripts were imported into MATLAB 2017b and processed using the recurrence quantification analysis (for guidelines and further information see Knight et al. [28] and Marwan et al. [53]) and the topic modelling toolbox based on Steyver’s and Griffith, respectively [44]. Self-reports, collected after the group activity, were used to assess the degree to which characteristics of group interactions explain the quality of participation and social support. The script for the RQA analysis as well as exemplar data are provided in the Additional files 1 and 2. The MATLAB scripts for the LDA analysis can be found online.^2^

## Data analysis

### Observational data and self-reports

We applied the Generalised Estimating Equations (GEES) approach in order to examine the relationship between group level RQA outcome measures and participants’ perceived quality of participation and social support (SPSS 24; IBM). GEEs are an extension of general linear model for analysing outcomes that violate the assumptions that are typically required e.g. non-normally distributed outcomes. Furthermore, the GEEs method has been recommended for use with clustered data and with datasets that are small in size and thus low in power [54, 55]. Because our data were not normally distributed and relatively small in size, the GEE is deemed as an ideal tool to test our theoretical assumption. Variables were tested for multicollinearity and the results were found to be below the advised degree for linear dependencies [56, 57]. Two GEEs were modelled: one examined the relationship between the RQA (stability, determinism, and complexity) and perceived quality of participation (i.e. outcome variable); the other examined the relationship between the RQA (stability, determinism, and complexity) and perceived social support.

### Qualitative data

Emerging groups of semantically related words were clustered into topics, for which the narrative structure was developed by two independent researchers who met throughout the data processing to identify, discuss, and resolve any inconsistencies in theme interpretation.

## Results

### Observational data and self-reports

The recurrence quantification analysis was carried out on a sequence of 157 (*SD* = 67) dyadic team interactions per 30-min group discussions. On average, groups revealed low levels of recurrence (*mean* =11.52; *SD* = 4.48), low to medium levels of predictability (*mean* = 35.73; *SD* = 10.42) and medium to high levels of complexity of sequences of group interactions (*mean* = 67.11; *SD* = 0.19). For the self-reports, groups had a mean level of perceived quality of participation and social support of 5.68 (*SD* = 0.61) and 4.78 (*SD* = 0.61), respectively. As illustrated in Table 1, there was no significant association between the quality of participation and the RQA. However, there was a significant positive association between group-level stability and perceived social support, as well as a significant negative association between perceived social support and the level of predictability in sequences of group interactions.

**Table 1:** Findings based on the Generalised Estimating Equations

Outcome	QIC	Parameter	B (SE)	95% CI		Wald	*p*
Variable				Lower	Upper	Chi-Square	
Quality of participation	13.433	(Intercept)	1.739 (.055)	1.630	1.847	992.734	.001
		Stability	.010 (.005)	−.001	0.022	3.170	.07
		Determinsim	−.002 (.003)	−.009	.005	.240	.62
		Complexity	−.086 (.205)	−.490	.317	.175	.67
Social support	11.215	(Intercept)	1.442 (.053)	1.336	1.547	716.971	.001
		Stability	.022 (.0063)	.009	.034	5.465	.001
		Determinsim	−.008 (.0033)	−.014	−.001	1.137	.01
		Complexity	.209 (.1961)	.593	1.137	1.137	.28

### Qualitative data

**Fig. 4:**
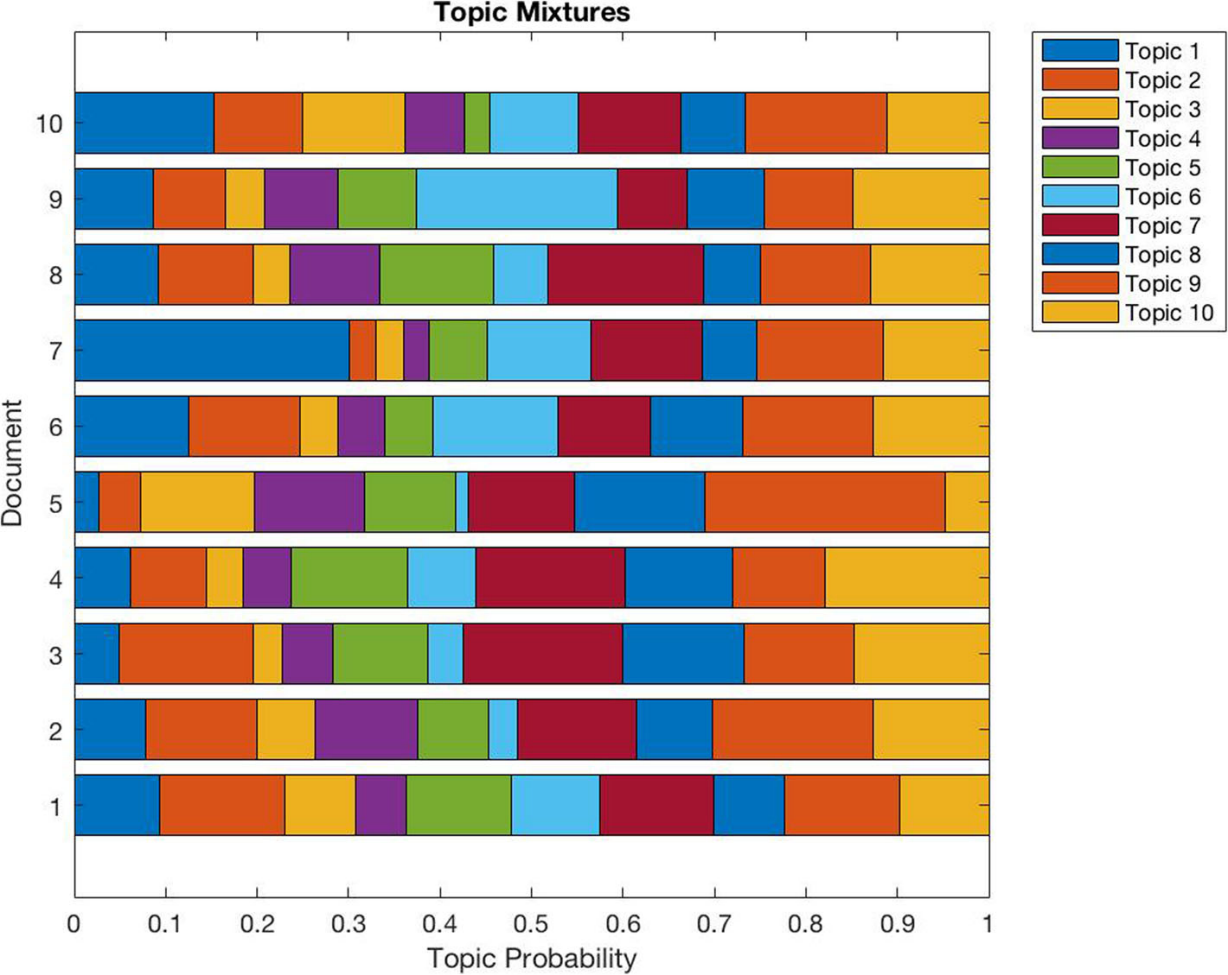
Topic probability for the first 10 documents

**Fig. 5:**
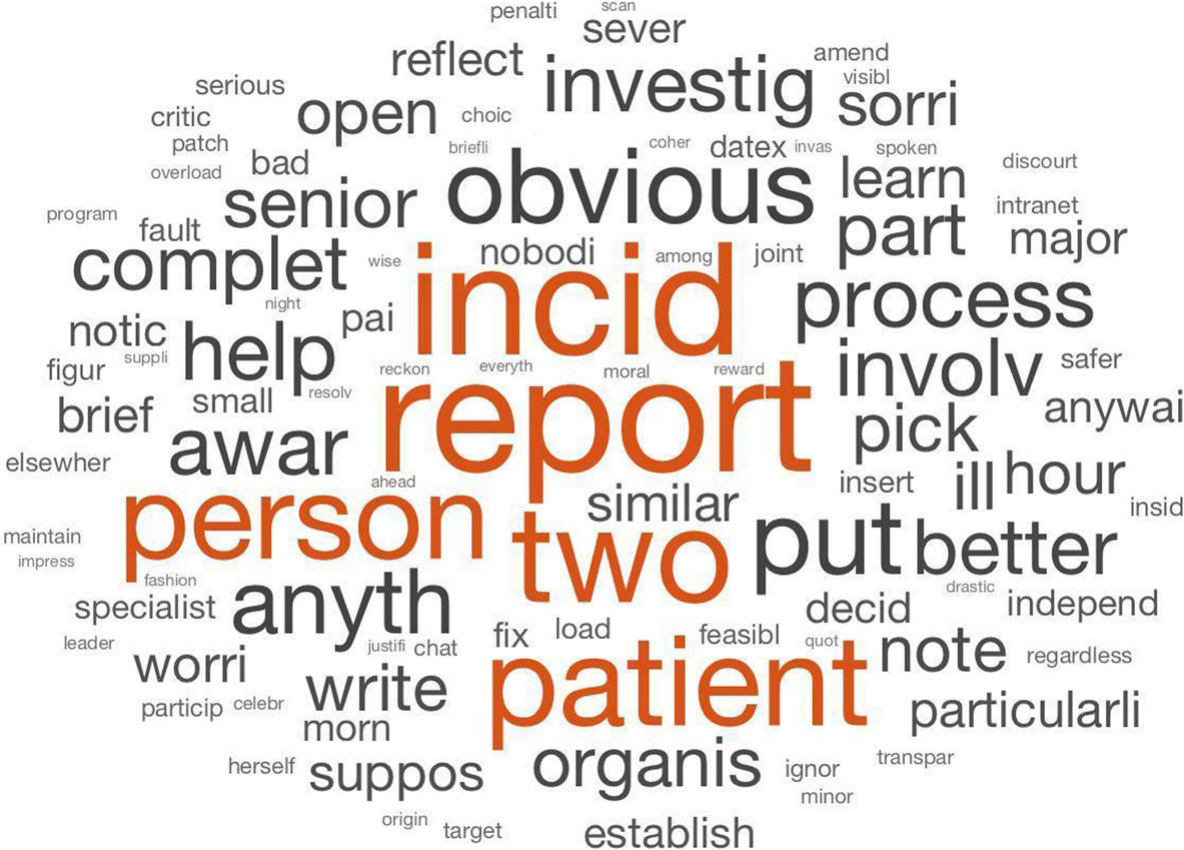
Word cloud created using the LDA approach

Results from the topic modelling method suggested that the data were best represented by fifteen topics. Figure 4 provides an illustration of the first ten topics for the first ten documents. Figure 5 shows the word cloud as visual illustration produced alongside the analysis. The first four topics are illustrated in the table below (cf. Table 2). Conversations addressed themes such as (1) areas and concerns related to the management of patient incidents; (2) the responsibilities among team members, including nurses and doctors; (3) the importance of a good internal team environment; and (4) the hospital culture. Although words within topics are low in probability, they remain within a reasonable range compared to other small group research studies [58].

**Table 2:** Word distributions of the first four topics with the highest probability (p) distribution

*Emerging Topics*							
Patient incident management	*p*	Doctor-Nurse responsibility	*p*	Team environment	*p*	Culture	*p*
“report”	0.086	“nurs”	0.112	“kind”	0.063	“culture”	0.057
“check”	0.063	“doctor”	0.044	“team”	0.041	“act”	0.049
“patient”	0.054	“audit”	0.038	“well”	0.034	“staff”	0.047
“inform”	0.028	“error”	0.032	“meet”	0.025	“prioriti”	0.043
“change”	0.023	“implement”	0.024	“group”	0.023	“learn”	0.025

## Discussion

This study examined group interactions and communication processes related to healthcare management and patient safety, with the aim to understand the nature of group interactions related to perceived quality of participation and social support, as well as to examine conversations around healthcare management and patient safety decisions using signal processing and machine learning technologies. Audio and video recordings of teams discussing healthcare management and patient safety were analysed using the RQA and the topic modelling approach.

Our findings revealed that group interactions were characterised by low stability, medium levels of predictability and medium to high complexity. The structural stability of interactions was positively related to perceived social support, while the predictability of sequences of interactions was negatively related to perceived social support. The quality of participation and perceived social support were illustrated by behavioural exemplars such as the encouragement of expression of views and opinions, showing respect for individual differences, and discouraging personal insults [2]. Concurrently, team members showed stable sequences of knowledge integration, with some degree of determinism of interaction that was represented by giving and receiving social support to other team members. While the interactions between members in a systematic and repetitive fashion allowed for an increased perception of participation and social support, the perception became negative when interactions occurred in a predictable manner. One reason for the latter could be that social support should be provided repeatedly but in consideration of the situational awareness necessary to provide support to the right person at the right time. However, if interactions become predictable, it might not lead to the same perception of support but to a common response pattern (e.g. person A always interacts with person B irrespective of content).

A practical example taken from these findings includes the scenario of optimising patient safety in operating theatres. During the simulations, various stakeholders provided input of their expertise to relevant factors in the simulation case, such as addressing systems organisation, factor analysis, employment practices and root causes. There was high variability in the suggestions and proposed solutions. Whilst the input was appropriate for each case, the variability was associated with the degree of encouragement needed to recognise team members’ contribution. For example, team members expressed different views about the priority to assign to practical tasks within the scenario, e.g. hiring the most appropriate surgeon versus general safety training for operating theatre staff. These conversations were reflected in a mix of stable patterns of group interactions, which, in previous research, have been associated with enhanced communication processes [28, 35]. Data from the current study add to these findings by demonstrating that the results of the RQA were associated with participants’ perceived social support. This is a novel finding which shows that the RQA indices have predictive power related to perceived quality indices of teamwork.

Spoken language data revealed points of discussions in real-life settings including the management of patient safety events; the responsibilities of team members, such as nurses and doctors; and an emphasis on a good internal team environment as well as the hospital culture. Similar to previous studies [59, 60], our data showed that that efficient healthcare management and patient safety is believed to consist of teamwork and effort that is reflected in clear roles and responsibilities, and that a culture of engagement and trust is essential for health systems to succeed in an era of accountable care.

Furthermore, findings are novel in that they highlight the role-play exercise as an appropriate environment to hold discussions about patient safety decisions and healthcare management. Topics identified as part of our set up, and in our findings, closely resemble the material used for this simulation. That is, the scenario of two serious incidents presented led to discussions around incident management, while the guideline on effective teamwork practices created discussions around team environment. As such, results are encouraging in that they could be used as a baseline for training and assessing patient safety decisions and healthcare management, and the simulation material as a tool of theory building around healthcare management and patient safety policy development, as well as for building practical applications that address sensitive and high-risk contexts such as serious incident management.

### Methodological consideration and implications for further research

Future studies could provide further guidelines on how to efficiently implement signal processing in this, and other, contexts. For instance, studies could address the degree to which the groups’ stability, predictability, and complexity are related to task accomplishment and objectively rated performance quality [61]. Thus, using standardised measures [50] alongside objective indices of teamwork and quality to examine patterns of cooperative inquiry and joint decision-making would facilitate a better understanding of the relationships and predictive tendencies between group interactions, perceived teamwork, observable behaviour, and team-level outcomes.

Related to ML techniques, the LDA, since being first developed by Blei et al. [62], has been subjected to extensions, such as the “topics over time” topic modelling approach [63], which uses time-stamps in texts to identify and cluster topics, or the “author-topic” modelling method [64] that links and clusters the words of each person to generate content in a group discussion. This approach would help to further understanding of the degree and content of each member’s contributions. While these methods were not of immediate relevance to our study since we examined the general feasibility of LDA as one of the fundamental ML methods to explore team-level conversations on patient safety and healthcare management, future studies could apply alternative approaches to address how discussion and decision-making change over time, and the degree to which each member’s input plays a role in the decision-making process.

### Limitations

Some limitations should be noted. We used self-reported data to understand the perceived quality of participation and social support rather than more objective ratings of quality of teamwork and performance outcomes (e.g. quality of problem solving through expert evaluation). To better assess the conditions for effective group interactions, future studies could obtain expert opinions in order to confirm the usefulness of the RQA as an attribute of teamwork and quality.

We used a simulated scenario to explore group interaction and conversations about patient safety and healthcare management. This method, while high in internal validity allowing for a scientifically robust establishment of the relationship between interactions and perceived quality of teamwork, has limited external validity, since everyday settings (e.g. board meetings) addressing patient safety incidents vary in duration, expertise, and content addressed. We also acknowledge that the role-play activity may have influenced the process and sequences of group interactions itself. While it was emphasised that participants should not feel limited by the role they were assigned to, and that they were welcome to bring in their own experience into the group discussion, future studies are encouraged to replicate this form of discussion in a real healthcare team environment in order to test the RQA application in real-life settings.

Lastly, while the results based on the LDA are in alignment with related studies in healthcare and management, results could be compared with e.g. positive and negative health-related practices, and with focus on potential policy recommendations and their implementations. For instance, by comparing the type of content addressed around serious incidents and by assessing the impact of final agreements on the quality of patient safety would be, although outside the scope if this research, a valuable further avenue of research. Thus, future studies are encouraged to carry out a robust validation, where themes from the ML technique are benchmarked against a range of health management practices established in real healthcare settings.

## Conclusion

Improving the quality of patient care requires the expression and coordination of specialist knowledge between team members, team members to support each other during the process of decision-making. The primary purpose of this study was to explore effective healthcare management and patient safety through the lens of group interaction and communication processes, and by using signal processing and ML techniques to analyse the data. Key findings show that stable yet complex team interactions are related to higher perceived social support. Moreover, conversations about patient safety and healthcare management addressed the reporting and handling of patient safety events, clear roles and responsibilities between team members, the importance of a good internal team environment, and the overall need to change the hospital culture towards commitment and trust. The research on appropriate management of serious incidents is still in its infancy and establishing further insights into how patient safety could be increased is an important aspect of healthcare. Future studies are, therefore, encouraged to apply both methods to determine further actions needed to improve healthcare service and delivery. Overall, findings contribute to examining and understanding the feasibility and effectiveness of both signal processing and ML techniques to analyse healthcare management practices, and offer a new avenue to theoretical developments that build on the enhanced use of data.

## Additional files

Additional file 1:  RQA Matlab script. (M 1 kb)

Additional file 2: RQA exemplar data. (MAT 236 bytes)

## Data Availability

The data that support the findings of this study are not publicly available. Because of the nature of the informed consent and ethical restrictions, data distribution is not permitted. However, we have provided MATLAB scripts and sample data sets of coded group interactions in the Additional files 1 and 2.

## Abbreviations

%DET: Percentage of determinism
ENTR: Entropy
LDA: Latent Dirichlet Allocation
ML: Machine learning
P: Probability
R: Correlation coefficient
RQA: Recurrence quantification analysis
RR: Recurrence rate

## Footnotes

1 The scripts can be downloaded here: http://psiexp.ss.uci.edu/research/programs_data/toolbox.htm and https://uk.mathworks.com/help/textanalytics/index.html?s_tid=CRUX_lftnav

2 http://psiexp.ss.uci.edu/research/programs_data/toolbox.htm and https://uk.mathworks.com/help/textanalytics/index.html?s_tid=CRUX_lftnav
